# Novel Retinoic Acid Receptor Alpha Agonists for Treatment of Kidney Disease

**DOI:** 10.1371/journal.pone.0027945

**Published:** 2011-11-18

**Authors:** Yifei Zhong, Yingwei Wu, Ruijie Liu, Zhengzhe Li, Yibang Chen, Todd Evans, Peter Chuang, Bhaskar Das, John Cijiang He

**Affiliations:** 1 Department of Medicine/Nephrology, Mount Sinai School of Medicine, New York, New York, United States of America; 2 Department of Pharmacology and Systems Therapeutics, Mount Sinai School of Medicine, New York, New York, United States of America; 3 Department of Molecular Biology in Surgery, The Weill Cornell Medical College, New York, New York, United States of America; 4 Department of Nephrology, Ruijin Hospital of Shanghai Jiantao University and Longhua Hospital of Shanghai University of Traditional Chinese Medicine, Shanghai, China; 5 Department of Radiology, Shanghai East Hospital of Tongji University, Shanghai, China; Florida International University, United States of America

## Abstract

Development of pharmacologic agents that protect podocytes from injury is a critical strategy for the treatment of kidney glomerular diseases. Retinoic acid reduces proteinuria and glomerulosclerosis in multiple animal models of kidney diseases. However, clinical studies are limited because of significant side effects of retinoic acid. Animal studies suggest that all trans retinoic acid (ATRA) attenuates proteinuria by protecting podocytes from injury. The physiological actions of ATRA are mediated by binding to all three isoforms of the nuclear retinoic acid receptors (RARs): RARα, RARβ, and RARγ. We have previously shown that ATRA exerts its renal protective effects mainly through the agonism of RARα. Here, we designed and synthesized a novel boron-containing derivative of the RARα-specific agonist Am580. This new derivative, BD4, binds to RARα receptor specifically and is predicted to have less toxicity based on its structure. We confirmed experimentally that BD4 binds to RARα with a higher affinity and exhibits less cellular toxicity than Am580 and ATRA. BD4 induces the expression of podocyte differentiation markers (synaptopodin, nephrin, and WT-1) in cultured podocytes. Finally, we confirmed that BD4 reduces proteinuria and improves kidney injury in HIV-1 transgenic mice, a model for HIV-associated nephropathy (HIVAN). Mice treated with BD4 did not develop any obvious toxicity or side effect. Our data suggest that BD4 is a novel RARα agonist, which could be used as a potential therapy for patients with kidney disease such as HIVAN.

## Introduction

Glomerular kidney disease is a major cause of End-Stage-Renal-Disease (ESRD) in the United States [1]. Treatment options for these diseases are scarce. Steroids and immunosuppressive medications are first-line treatments for glomerular diseases. However, resistant cases are frequently observed and side effects from the treatment are multiple. Treatment of glomerular diseases with angiotensin converting enzyme inhibitors or angiotensin receptor blockers reduces proteinuria and slows the progression of kidney disease. However, they only provide partial protection. Therefore, it is important to identify new treatment targets or regimes.

HIV-associated nephropathy (HIVAN), characterized by collapsing focal segmental glomerulosclerosis (FSGS), is a leading cause of kidney disease in young African Americans [2]. Although suppression of viral replication with antiretroviral therapy alters the course of the kidney disease, a specific treatment for this disease is not available, and many patients with HIVAN still progress to ESRD [3]. Podocyte injury is a major cause of kidney diseases, including HIVAN. Podocyte dedifferentiation and proliferation is a unique feature observed in HIVAN [4]–[5]. HIV-infected podocytes lose differentiation markers including synaptopodin and WT1 [6]. *In vitro*, HIV infection causes podocyte proliferation and dedifferentiation through activation of MAPK and Stat3 pathways [7]. Transgenic mice expressing HIV-1 gene in podocytes develop kidney disease similar to HIVAN [8]. Therefore, prevention or reversal of podocyte injury is an important strategy to treat HIVAN. So far, no drugs are available to specifically prevent or reverse podocyte injury as a treatment option for glomerular diseases.

Retinoids are derivatives of vitamin A and have multiple cellular functions including inhibition of proliferation, induction of cell differentiation, regulation of apoptosis, and inhibition of inflammation [9]. During kidney development, retinoic acid affects tubulogenesis and nephron number [10]. In addition to their established benefits for treatment of a variety of cancers, retinoids have been shown to protect against renal injury in multiple experimental models of kidney diseases [11]. In rat models of acute and chronic mesangioproliferative glomerulonephritis, retinoids preserve renal function, decrease albuminuria, and reduce glomerular and tubular damage [12] [13]. In a rat model of puromycin aminonucleoside-induced nephrosis, retinoids prevent proteinuria by protecting podocytes from injury [14] [15]. Treatment with isotretinoin significantly reduces glomerular damage in rats with chronic glomerulonephritis [16]. ATRA treatment also reduces lymphoproliferation and glomerulonephritis in MRL/lpr mice [17]. The protective effects of retinoids have also been reported in mice with diabetic nephropathy [18] and in an antibody-mediated model of podocye injury [19]. ATRA can restore the expression of podocyte differentiation markers including nephrin, podocin, and synaptopodin in vivo [19]. Recently, we found that ARTA reduces proteinuria and glomerulosclerosis in the HIV-1 transgenic mouse (Tg26), an animal model of HIVAN [20], by inhibiting cell proliferation and restoring differentiation markers in HIV-infected podocytes [20]. In addition, ATRA was shown to inhibit HIV-induced podocyte proliferation through activation of MAPK phosphatase 1 (MKP1) leading to the inhibition of MAPK phosphorylation [21]. These studies provide a strong support for using retinoic acid to treat glomerular diseases where podocytes injury is a prominent factor in disease pathogenesis. A phase II clinical trial is currently ongoing to examine the effect of ATRA in patients with glomerular diseases with podocyte injury including steroid-resistant minimal change disease, FSGS, and HIVAN (NIDDK website). However, clinical use of ATRA is challenging due to its side effect profile, which includes differentiation syndrome [22], depression and psychosis, severe acne, dryness of skin and mucosal membranes, inflammatory bowel diseases, and teratogenicity.

Our recent studies suggest that retinoic acid improves kidney glomerular diseases and protects podocytes from injury by activating retinoic acid receptor-α (RARα) [23]. We found that Am580, a RARα-specific agonist, induces podocyte differentiation in vitro and attenuates kidney injury of Tg26 mice in vivo [23]. Knockout of RARα aggravates kidney injury in Tg26 mice. Taken together, these data strongly support using RARα agonists for the treatment of kidney disease. It is critical to identify new specific agonists for RARα that possess the therapeutic function, but eschew the undesirable side effects of retinoic acid.

Several studies suggest that the toxicity of structural analogs of retinoic acid may be considerably reduced if a heterocyclic ring is incorporated. For instance, the presence of a substituted chromene moiety in the retinoic acid backbone, such as in the oxaretinoids, considerably reduced the toxicity of the compounds [24] [25] [26] [27] [27]. Based on these findings, we have synthesized a novel RARα agonist, BD4, and characterized its efficacy for the treatment of kidney disease in Tg26 mice.

## Results

### 1. Synthesis of a heteroaraotinoid—BD4

**Figure 1:**
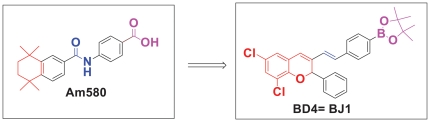
Synthesis of RARα agonist BD4. We designed and synthesized a novel RARα agonist, BD4, by making a bridge of a functional group (trans double bond) between hydrophobic chromene rings and benzoic acid or acid isosteres. The functional group was designed not only to increase the efficacy but also to reduce the toxicity of compounds. These modifications also increase the specificity for retinoic acid receptor isoforms. Characteristic features of BD4 are as follows: a) Our compound BD4 is oxaretinoid; b) Incorporation of oxygen heteroatoms to replace one of the gem-dimethyl groups in the tetrahydronaphthalene ring of Am580 decrease the toxicity by retarding metabolic oxidation; c) To protect against protease-mediated hydrolysis of amides bonds found in Am580, we introduced trans-double bond as amide isosteres in BD4, which is more resistant to proteolysis; d) Substitution of boronic acid and ester in BD4 in place of acids generates a more biologically active molecular frameworks, which allows BD4 to interact with a target protein through both hydrogen bonds and covalent bonds. This interaction is predicted to produce more potent biological activity.

**Figure 2:**
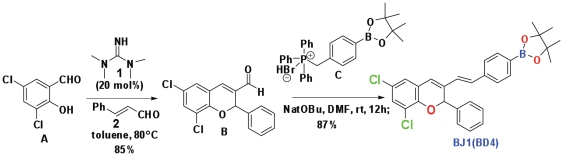
Reaction scheme to synthesize BD4. Compound B was synthesized from compound A by organocatalytic domino oxa-Michael/aldol reaction using organocatlyst 1. Compound B was further derivatized to compound BD4 by using Wittig reactions using Compound C as Wittig salt.

To generate BD4, we made the following modifications to Am580. We introduced an acid-bioisosteres group—boronic acid and a 3-substituted-2-phenyl-2H-chromene derivatives to protects isomerization of alkene spacers at 9, 13, 15 position of ATRA. No prior description of the biological effects of these compounds has been reported. The structural comparison between BD4 and Am580 is shown in Figure 1. Synthesis of BD4 is summarized in Figure 2. Our compound BD4 is unique as this is the first boronic-acid containing retinoids. By incorporating oxygen heteroatoms and replacing one of the gem-dimethyl groups in the tetrahydronaphthalene ring of Am580 we decreased the potential toxicity of Am580 by retarding metabolic oxidation. To avoid protease-mediated hydrolysis and improve the stability of the BD4 we introduced amide isosteres trans-double bonds in place of amide bonds in Am580.

All retinoids and synthetic heteroarotinoids reported to date contain acid groups. The use of boron atoms in pharmaceutical drug design has a high potential for discovery of new biological activities [28]. The boron atom has a vacant orbital and inter-converts with ease between the neutral and anionic hybridization states, which generates a new stable interaction between the boron atom and a donor molecule through a covalent bond [29]. Therefore, it is anticipated that boron atoms introduced into biologically active molecular frameworks could interact with a target protein not only through hydrogen bonds but also through covalent bonds, and this interaction might produce potent biological activity, a concept that is well supported by the literature [30]. Among various synthetic boron compounds, considerable attention has been placed on boronic acid-containing peptides such as Velcade and DPP-IV inhibitors [31].

We screened our small focused library. Unlike the commonly employed approach of high-throughput screening of large libraries to identify lead compounds that elicit a desired phenotypic effect, we were utilizing a Limited Rational Design approach, which means rather than screening 10,000-100,000 arrayed compounds we generated a small library of only about 40-50 to identify lead compounds. We used the synthesis strategies for boron-containing unnatural amino acids and peptides and heterocycles as described previously [32] [33] [34] [35] [36].

### 2. BD4 binds to RARα with a higher affinity than ATRA and Am580

**Figure 3:**
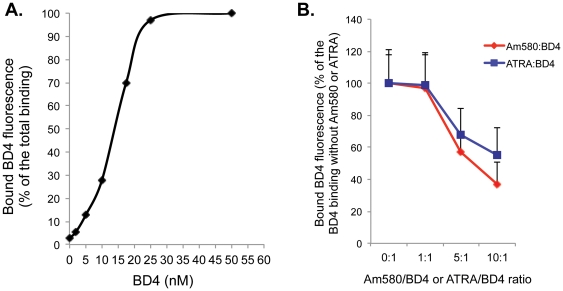
RARα binding assay. A fluorometric binding assay was performed as described in the method. **A.** The scatter curve was built between different concentrations of BD4 and percentage BD4 binding to calculate the KD. A representative curve is shown from four independent experiments. KD = 14±5 nM, n = 4. **B.** Competitive binding assays were performed between BD4 and Am580 or BD4 and ATRA. No significant inhibition of binding was observed at 1∶1 ratio. However, a 40% inhibition of BD4 binding was observed when Am580 to BD4 or ATRA to BD4 ratio was increased to 5∶1. These data suggest that the affinity of BD4 binding to RARα is higher than Am580 and ATRA.

First, we confirmed that BD4 binds specifically to RARα. Since we were unable to label BD4 directly by either tritium or fluorescence tagging, we used a modified fluorometric assay that has been previously described [37] to assess BD4 binding to RARα. Using this fluorometric binding assay we confirmed that BD4 binds to RARα with an affinity of 14±5 nM (Figure 3A), which is in the range of what has been reported previously for Am580 [38]. We also performed competitive binding assays for BD4 and Am580 and for BD4 and ATRA. We found that 5-to-10 fold of Am580 or ATRA was required to induce a 40–50% competitive inhibition of BD4 binding to a GST-tagged RARα. This confirms that BD4 binds to RARα with a higher affinity than Am580 or ATRA (Figure 3B).

### 3. BD4 is not toxic to podocytes in culture

**Figure 4:**
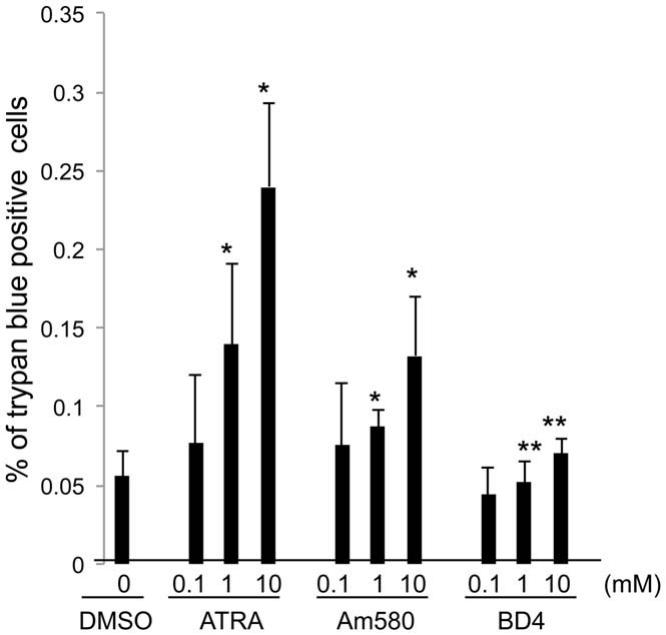
Cell toxicity assay. Mouse podocytes cultured at 37°C for 10 days and then treated with ATRA, Am580, or BD4 at the different concentrations for 24 hours in serum free medium. Cell death was quantified by trypan blue staining, n = 6, *p<0.01 compared to cells treated with DMSO, **p<0.05 compared to cells treated with either ATRA or Am580 at corresponding concentrations.

Next, we compared the cellular toxicity among ATRA, Am580, and BD4 in podocytes by trypan blue staining to determine cell death. Podocytes were cultured with ATRA, Am580, and BD4 at different concentrations for 24 hours in serum free medium. Cell death was quantified by trypan blue staining. We found that BD4 had significantly lower cell toxicity than ATRA and Am580 at 1 µM and 10 µM (Figure 4).

### 4. BD4 promotes podocyte differentiation in vitro

**Figure 5:**
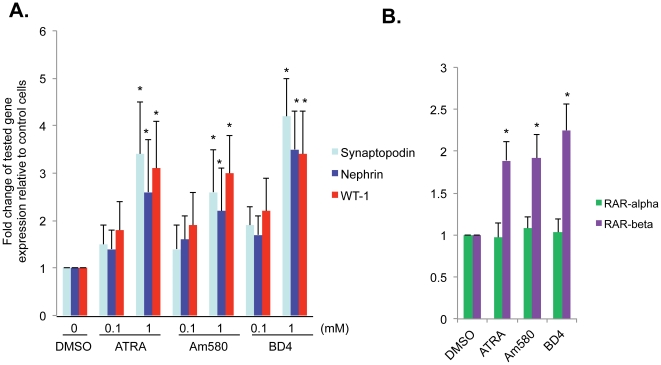
Expression of podocyte differentiation markers in vitro. **A.** Mouse podocytes cultured at 37°C for 10 days and then stimulated with either ATRA, or Am580, or BD4 at 0.1 µM and 1 µM for 24 hours in serum free medium. Total RNA was extracted and analyzed by real-time PCR to determine the expression of podocyte differentiation markers (synaptopodin, nephrin, and WT-1). **B.** Podocytes stimulated with either ATRA, or Am580, or BD4 at 1 µM for 24 hours in serum free medium. Real-time PCR analysis of RARα and RARβ expression were performed. The ratios of these genes normalized to GAPDH expression are expressed. N = 4, p*<0.01 compared to cells treated with DMSO.

To assess the biologic effect of BD4 on podocyte differentiation, we used a conditionally immortalized murine podocyte cell line which has been used extensively to study podocyte differentiation in vitro [39]. These podocytes express a temperature-sensitive T antigen, which is stable at 33°C and inactive at 37°C. T-antigen activates cell proliferation at 33°C. At 37°C, T-antigen is inactivated and cells grow slowly and express podocyte differentiation markers to some extent (partial differentiation). Our previous studies suggest that ATRA can induce further differentiation of these podocytes at 37°C by stimulating the expression of podocyte differentiation markers including synaptopodin, nephrin and Wilm's Tumor 1 (WT1). Thus, we compared the effects of BD4, ATRA, and Am580 on the expression of podocyte differentiation markers in these partially differentiated podocytes. We found that BD4 significantly stimulates the expression of synaptopodin, nephrin and WT1 to levels similar what has been previously observed with ATRA or Am480 at the same concentration (1 µM) (Figure 5A). As a control, we also examined the expression of RARα and RARβ in these cells. We found that BD4 increases the mRNA of RARβ without changing the expression of RARα, which is similar to what we had observed previously with ATRA and Am580 [23] (Figure 5B). It is well known that RARβ, but not RARα, is a target gene of retinoid acid response element (RARE) [40].

### 5. Determine the effects of BD4 in HIV-1 transgenic mice (Tg26)

We previously demonstrated that treatment of Tg26 mice with Am580 ameliorates kidney disease. To determine the effect of BD4 on the development of kidney disease in Tg26 mice, we treated Tg26 mice with either vehicle or BD4 by daily gavages from age of 4 weeks, which is approximately the age when Tg26 animals develop significant proteinuria. After 6 weeks of treatment, these mice were sacrificed. Serum was collected and blood urea nitrogen (BUN), as a marker of glomerular function, was measured. Urine samples were collected for determination of albumin and creatinine and albumin/creatinine ratio was calculated. Kidney samples were fixed then paraffin-embedded. Glomeruli were isolated from kidneys. mRNA levels of markers for differentiated podocytes were determined by real-time PCR using total RNA extracted from isolated glomeruli.

**Figure 6:**
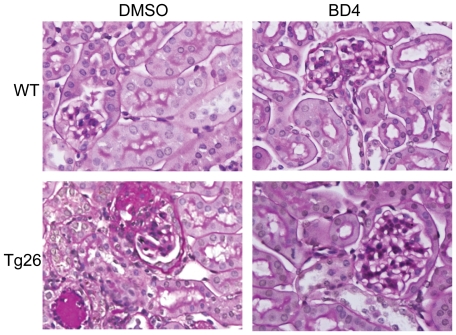
Kidney histology. Both Tg26 and WT mice were fed with either control vehicle or BD4 by daily gavage from 4 to 12 weeks of age. Mice were sacrificed at 12 weeks of age and the kidneys were removed for histology analysis. The representative H&E stained sections from six mice in each group are shown.

**Figure 7:**
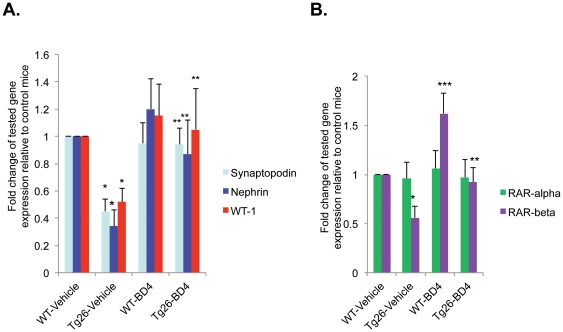
Expression of podocyte differentiation markers in vivo. Glomeruli were isolated from both WT and Tg26 mice treated with vehicle or BD4 for 6 weeks. Total RNA was isolated from the glomeruli for real-time PCR analysis for the podocyte differentiation markers including synaptopodin, nephrin, and WT-1 (**A**) as well as for RARα and RARβ (**B**). The ratio of these genes normalized to GAPDH was calculated. n = 6, *p<0.01 compared to vehicle-treated WT mice (WT-vehicle), **p<0.05 compared to vehicle-treated Tg26 mice (Tg26-vehicle), ***p<0.05 compared to vehicle-treated WT mice (WT-vehicle).

We found that BD4 significantly reduced BUN and proteinuria in Tg26 mice compared to those treated with vehicle (Table 1). We also confirmed that kidney injury (glomerulosclerosis and tubule-interstitial fibrosis) was also significantly improved with BD4 (Table 1 and Figure 6). In addition, BD4 treatment restored the expression of podocyte-specific markers—synaptopodin, nephrin and WT1 (figure 7A). Consistent with our previous findings [23], BD4 also increased mRNA levels of RARβ in both WT and Tg26 glomeruli while RARα expression unaffected (Figure 7B). Taken together these data suggest that BD4 attenuates kidney injury and improves podocyte differentiation in Tg26 mice. These beneficial effects of BD4 in Tg26 mice are similar to those we had previously reported for Tg26 mice treated with ATRA and Am580.

**Table 1:** Effects of BD4 on mouse body weight, BUN, proteinuria, and kidney histology.

	Body Weight	BUN (mg/dl)	Albumin/Cr ratio	GS index	Podocyte Hypertrophy	Tubular casts/cysts
WT + Vehicle	22.6±0.8	24.6±2.4	0.12±0.10	0	0	0
Tg26 + Vehicle	21.6±1.8	39.6±3.4	3.34±1.56	18.5±11.1	1.2±0.8	9.4±5.6
WT + BD4	22.2±1.2	25.4±3.2	0.18±0.22	0	0	0
Tg26 + BD4	21.3±2.3	28.5±3.8*	0.34±1.5*	2.7±0.78*	0.3±0.5*	1.4±0.9*

Tg26 and the WT littermates were treated with BD4 or control DMSO for 6 weeks. Body weight was recorded when mice were sacrificed. Blood samples were collected for measurement of BUN and urine samples were collected for determination of albumin and creatinine. Histology of these kidneys was analyzed as described in the method, n = 6,

*p<0.01 compared to Tg26 mice treated with vehicle.

## Discussion

The treatment of kidney glomerular disease is challenging. Many lines of evidence suggest that retinoic acid can improve kidney injury in animal models of kidney disease [9]. However, long-term clinical use of retinoids in patients with kidney disease is difficulty due to significant side effects. A phase II clinical study approved by NIH has not been completed likely because of recruitment issues (NIDDK website). Recently, we have shown that the beneficial effects of ATRA for treatment of kidney disease in HIV1 trangenic mice are mediated through the activation of RARα [23]. RARα agonists can reduce proteinuria and glomerulosclerosis and protect podocytes from injury [23]. Our previous data suggest that Am580 can improve kidney injury in HIV-1 transgenic mice [23]. It is known that Am580 may have less toxicity than ATRA because most side effects of ATRA are likely mediated by activation of RARγ [41] [42]. However, significant side effects are still reported with Am580 [42]. Therefore, further discovery of better RARα agonists are warranted.

In this study, we have established the efficacy of a novel synthetic retinoid, BD4, in ameliorating the development of kidney disease in Tg26. BD4 should have a more favorable side-effect profile based on its molecular structure. The low cellular toxicity in cultured podocytes and the lack of increase in morbidity and mortality in the BD4-treated animals suggest that BD4 is not toxic. However, the effect of BD4 on other known side effects of retinoids including teratogenicity, dyslipidemia, depression, dry skin, and inflammatory bowel disease will need to be further investigated.

Although our data suggest that RARα agonists reduce kidney injury in HIV-1 transgenic mouse, a model for HIVAN. Based on previous studies, we believe that RARα agonists would also protect against podocyte injury in other forms of glomerular diseases. Our unpublished data suggest that Am580 also protects against diabetic nephropathy in mice.

Based on these findings, we conclude that BD4, a new RARα agonist, induces podocyte differentiation in vitro and improves proteinuria and glomerulosclerosis of HIV-1 transgenic mice in vivo. BD4 might also protect against other forms of glomerular diseases such as FSGS and diabetic kidney disease. The toxicity and side-effect profile of BD4 will need to be investigated further prior to clinical trials in human subjects.

## Methods

### 1. Synthesis of BD4

All reagents were purchased from commercial sources unless otherwise indicated. Products were purified by column chromatography over silica gel. ^1^H NMR and ^13^C NMR spectra were recorded at 25 °C at 300 MHz and 75 MHz, respectively, with TMS as internal standard. Abbreviations for signal coupling are as follows: s, singlet; d, doublet; t, triplet; q, quartet; m, multiplet; br, broad. Column chromatography was performed using SiO2 (0.040 – 0.063 mm, 230 – 400 mesh ASTM) from Merck. Mass spectra were recorded on Varian MS mass spectrometer. To the stirred solution of aldehyde B (compound B in Figure 1, 400 mg, 1 mmol) and 4-(4,4,5,5-tetramethyl-1,3,2-dioxaboratophenyl)-methyl triphenylphosphonium bromide (compound C in Figure 1, 880 mg, g, 2 mmol) in anhydrous DMF was added and stirred. Sodium *tert*-butoxide (0.38 g, 0.003 mmol) was added to the solution at 0°C and allowed it to stir in RT for 12 h. The deep brown color reaction mixture was poured into water (20 mL) and extracted with ethylacetate (3×10 mL).The organic layer was washed with H_2_O, brined and dried over anhydrous Na_2_SO_4_ and filtered. Evaporation of the solvent followed by column chromatography on silica gel using 5% ethylacetate/hexane afforded pure olefin as yellow solid BJ1(BD4) (480 mg, 73%). Compound BD4 has the following spectral signature: ^1^H NMR (300 MHz, CDCl_3_): *δ* 7.77 (d, *J* = 9.0 Hz, 2H), 7.52-7.48 (m, 2H), 7.42 (d, , *J* = 9.0 Hz, 2H), 7.33 (dd, *J*_12_ = 3.0 Hz, *J*_13_ = 9.0 Hz, 3H), 7.13 (d, *J* = 3.0 Hz, 1H), 7.07 (d, *J* = 15.0 Hz, 1H), 7.01(d, *J* = 3.0 Hz, 1H), 6.74 (s, 1H), 6.51(d, *J* = 15.0 Hz, 1H), 6.40(s, 1H), 1.37(s, 12H); ^13^C NMR (75 MHz, CDCl_3_): *δ* 146.9, 139.4, 137.7, 135.5, 134.9, 131.4, 129.3, 129.2, 129.0, 127.9, 127.8, 126.5, 126.3,125.7, 125.0, 123.6, 122.7, 84.2, 77.6, 25.2 ESI MS: [M+H] 505.1352, Calcd 504.1430 for C_29_H_27_BCl_2_O_3_

### 2. RARα ligand binding assay

Our lead molecule BD4 contains a boronic acid group. For tritiation reaction, Ruthenium (Ru) and other organometallic catalysts are required to introduce tritium inside the phenyl ring system. Since the boronic-ester group is very prone to demetalation in the presence of organometalic catalysts and also it may undergo coupling reactions, it is technically difficult to label BD4 with tritium. Labeling of BD4 with a fluorescence tag is also problematic. Since BD4 is a small molecule, tagging it with a fluorescence marker has a high potential to interfere with its binding to RARα. To overcome these challenges, we used a modified version of a fluorometric binding assay that has been previously described [37]. First, we determined the fluorescence spectrum of BD4 in the range of excitation wavelength from 300 nm to 450 nm and emission wavelength from 350 nm to 500 nm. An emission peak of 480 nm was found at the excitation wavelength of 380 nm. There was very low baseline emission reading at this wavelength. A linear relationship was observed between the amount of BD4 and fluorescence emission at 480 nm. This allowed us to develop a fluorometric assays to assess BD4 binding to RARα. Purified RARα was generated using a bacterial expression system. First, RARα cDNA was subcloned into pGEX bacterial expression construct and GST-RARα fusion protein was purified from bacterial lysate using GST affinity columns as described previously [21]. For BD4 binding experiments, different concentrations of BD4 were mixed with 2×10^−7^ M of purified GST-tagged RARα at 37°C for 10 minutes and emission at 480 nm was measured with excitation of 380 nm. Since the addition of albumin, GST, or GST-RARα into BD4 solution did not change the fluorometric reading at baseline, we decided to precipitate GST-RARα-bound BD4 by glutathione beads and removed them by centrifugation. This allowed us to measure the fluorescence of unbound BD4 in the supernatant. Similar experiments were performed with GST as a non-specific control for BD4 binding. Based on these data, we were able to calculate the fraction of BD4 bound to RARα by subtracting the unbound BD4 (after precipitation) from total BD4 fluorescence (before precipitation). A scatter curve between BD4 concentrations and the percentage of RARα−bound BD4 was built to calculate the affinity of binding (KD).

Next, we determined the binding affinity of Am580 and ATRA to RARα. Since Am580 and ATRA do not have the same fluorometric activity as BD4 (Ex 380 nm and Em 480 nm), we performed competitive binding assays by incubating varying concentrations of either Am580 or ATRA to compete with BD4 for binding to GST-RARα ~ Fluorometric measurements at 480 nm were made to determine the percentage of BD4 bound to GST-RARα and estimate the relative affinity of RARα to BD4, Am580, and ATRA.

### 3. Podocyte cultures and in vitro studies

Podocyte cell culture: Conditionally immortalized murine podocytes are gifts from Dr. Peter Mundel (Massachusetts General Hospital, Boston). To permit immortalized growth, the culture medium was supplemented with 10 units/mL of recombinant mouse γ-interferon to induce the expression of T antigen and cells were cultured at 33°C (permissive conditions). To induce differentiation, cells were cultured on type I or collagen IV at 37°C without γ-interferon for at least 10 days. We confirmed the degradation of the T antigen under nonpermissive condition (37°C) by Western blot analysis. Partially differentiated podocytes at 37°C were used in all cell culture experiments. Cells were stimulated with either ATRA, or Am580, or BD4 at the different doses for 24 hours in the serum-free medium. Then, cells were harvested for total RNA isolation and real-time PCR analysis of podocyte-specific gene expression.

### 4. Animal studies

HIV-1 transgenic mice (Tg26) and their age-matched corresponding littermates (n = 6 per group including 3 male and 3 female mice in each group) were fed with either control vehicle or BD4 by daily garvage at a concentration of 0.3 mg/kg/day. The mice were fed with this compound everyday from age of 4 weeks to 10 weeks. Unrestricted food and water were provided throughout the duration of the experiment. The mice were euthanized at 10 weeks of age for blood, urine, and tissue collection by exposure to carbon monoxide. Body and kidney weight were recorded. All animal studies were performed according to the protocols approved by Institutional Animal Care and Use Committee at the Mount Sinai School of Medicine (GCO#06-1098).

### 5. Measurement of BUN, urine protein, and creatinine

Blood urea nitrogen (BUN) was measured by using a commercially available kit (Bioassay Systems, Hayward, CA). Urine albumin was quantified by ELISA using a kit from Bethyl Laboratory Inc. (Houston, TX, USA). Urine creatinine levels were measured in the same samples using QuantiChrom™ Creatinine Assay Kit (DICT-500) (BioAssay Systems) according to the manufacturer instruction. The urine albumin excretion rate was expressed as the ratio of albumin to creatinine.

### 6. Quantitative Histopathology

Mice were perfused with PBS containing 4% paraformaldehyde and kidneys were further fixed in 4% paraformaldehyde for 2 hours. Kidney tissue was embedded into paraffin. Kidney histology was examined after periodic acid-Schiff (PAS) staining. Glomerulosclerosis was scored as described previously by Dr. D'Agati [43]. Briefly, each specimen received a score for three parameters: percentage of collapsing glomerular sclerosis, percentage of tubular cysts or casts, and podocyte hypertrophy. The percentage of collapsing glomerulosclerosis was obtained by identifying the total number of glomeruli with any sclerosis and dividing this number by the total number of glomeruli seen. The percentage of tubular cysts or casts score was obtained by the number of tubules with either microcystic dilatation or filled with casts divided by the total number of tubular cross sections in a representative area. Finally, the degree of podocyte hypertrophy was scored as 0 (absence), 1+ (podocyte hypertrophy observed in less than 25% of all glomeruli), 2+ (podocyte hypertrophy observed in between 25–50% of all glomeruli), and 3+ (podocyte hypertrophy in greater than 50% of all glomeruli).

### 7. Isolation of glomeruli from mice for western blot and real-time PCR

Mouse glomeruli were isolated as described [44]. Briefly, animals were perfused with Hank's Buffered Salt Solution (HBSS) containing 2.5 mg/ml iron oxide and 1% bovine serum albumin. At the end of perfusion, kidneys were removed, decapsulated, minced into 1-mm^3^ pieces, and digested in HBSS containing1 mg/ml collagenase A and 100 U/ml deoxyribonuclease I. Digested tissue was then passed through 100 micron cell strainer and collected by centrifugation. The pellet was resuspended in 2 ml of HBSS and glomeruli were collected using a magnet. The purity of glomerular was verified under microscopy and by western blot analysis for podocyte specific markers including synaptopodin, nephrin, and WT-1.

### 8. Real-time PCR

Total RNA was isolated from kidney glomeruli of mice using TRIzol (Invitrogen). Real-time PCR was performed with a Roche Lightcycler and Qiagen QuantiTect One Step RTPCR SYBR green kit (Qiagen) according to the manufacturer's instructions. Pre-designed primer sets were obtained from Qiagen (GeneGlobe) for synaptopodin, nephrin, WT-1, RARα, RARβ, and GAPDH. The sequences of the primers are summarized in the Table 2. Light cycler analysis software was used to determine crossing points using the second derivative method. Data were normalized to housekeeping genes (GAPDH) and presented as fold increase compared to RNA isolated from WT animals using the 2^−^ 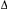 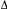 ^CT^ method.

**Table 2:** Sequences of the primers.

Gene name	sense	antisense
Synaptopodin	5′-GACACCGTTTCCTCTCCGC	5′-AGAAAAGCGTCAGACAGCAGT
Nephrin	5′- GTGCCCTGAAGGACCCTACT	5′-CCTGTGGATCCCTTTGACAT
WT-1	5′-GAGAGCCAGCCTACCATCC	5′-GGGTCCTCGTGTTTGAAGGAA
RARα	5′-CGCTCCGGACTCCGCTTTGG	5′-CCCTTGCAGCCCTCACAGGC
RARβ	5′-ACCGAATGGCAGCATCGGCA	5′-TCCCTCACAGGCGCTGACCC
GAPDH	5′-TGTTGCCATCAATGACCCCTT	5′-CTCCACGACGTACTCAGCG

### 9. Statistical Analysis

Data were expressed as mean ± standard deviation (*X*±SD). The unpaired T-test was used to analyze data between two groups. ANOVA was used for multiple group analysis and the comparison between the groups was further analyzed using Bonferroni correction. The renal scoring data was analyzed by using non-parametric Wilcoxon Signed Rank Test. Statistical significance will be considered when *p*<0.05.
